# Syndrome cardio-rénal: aspects épidémiologiques, à propos de 36 cas dans un service de cardiologie de Dakar

**DOI:** 10.11604/pamj.2017.28.58.10257

**Published:** 2017-09-21

**Authors:** Malick Bodian, Awa Thiaw, Simon Antoine Sarr, Kana Babaka, Fatou Aw, Aliou Alassane Ngaïde, Mouhamadou Bamba Ndiaye, Adama Kane, Modou Jobe, Alassane Mbaye, Maboury Diao, Moustapha Sarr, Serigne Abdou Ba

**Affiliations:** 1 Service de Cardiologie, Centre Hospitalier Universitaire Aristide Le Dantec, Dakar, Sénégal; 2 Service de Cardiologie, Hôpital Général de Grand Yoff, Dakar, Sénégal

**Keywords:** Syndrome cardio-rénal, epidemiologie, hypertension, insuffisance renale, Dakar, Cardiorenal syndrome, epidemiology, hypertension, renal failure, Dakar

## Abstract

**Introduction:**

Le syndrome cardio-rénal (SCR) est un trouble physiopathologique du cœur et des reins dans lequel une dysfonction chronique ou aiguë de l’un peut induire une dysfonction chronique ou aiguë de l’autre. En Afrique et au Sénégal en particulier l’incidence du syndrome cardio-rénal est mal connue. L’objectif de cette étude était d’apprécier la prévalence du SCR en milieu cardiologique.

**Méthodes:**

Nous avons mené une étude rétrospective incluant tous les insuffisants cardiaques ayant une altération de leur fonction rénale et hospitalisés entre avril 2010 et avril 2011 au service de cardiologie. Les données étaient analysées avec le logiciel statistique Epi-info 3.5.3.

**Résultats:**

Nous avons inclus 36 patients. La prévalence était de 3,7% avec une prédominance masculine (sex-ratio à 1,77) et un âge moyen à 56,9 ans [30-92]. Les antécédents étaient dominés par l’hypertension artérielle (52,77%) et le diabète (19,4%). Les principales étiologies étaient la cardiomyopathie hypertensive (39%) et l’insuffisance coronarienne (19,44%). La symptomatologie était dominée par la dyspnée (69,4%) et les œdèmes (50%). On notait une anémie (17 patients). La clairance moyenne (MDRD) à 46 ml/min. L’échocardiographie Doppler retrouvait majoritairement des troubles de la cinétique (89,3%), une dysfonction systolique VG (71%). Les 3 échographies rénales étaient normales. Six décès (16,7 %) étaient notés.

**Conclusion:**

Le syndrome cardio-rénal est une réalité et marque un tournant dans l’évolution de toute cardiopathie et néphropathie. Sa prévalence en milieu cardiologique sénégalais est faible. Des études prospectives et multicentriques sont nécessaires pour une meilleure évaluation au Sénégal.

## Abstract (en)

**Introduction:**

Cardiorenal syndrome (CRS) is a pathophysiologic disorder of the heart and the kidneys whereby acute or chronic dysfunction in one organ may induce acute or chronic dysfunction of the other. In Africa, particularly in Senegal, the incidence of cardiorenal syndrome is not accurately known. This study aimed to assess the prevalence of CRS in the Cardiology Department.

**Methods:**

We conducted a retrospective study including all patients with heart failure associated with alteration of renal function, hospitalized in the Cardiology Department between April 2010 and April 2011. Data were analyzed with the statistical software Epi-Info 3.5.3.

**Results:**

36 patients were included in the study. The prevalence rate was 3.7% with male predominance (sex-ratio 1.77) and an average age of 56.9 years [30-92]. Patients’ medical history was dominated by high blood pressure (52.77%) and diabetes (19.4%). The main etiologies were hypertensive cardiomyopathy (39%) and coronary heart disease (19.44%). The symptomatology was dominated by dyspnoea (69.4%) and edema (50%). 17 patients had anemia. The mean measured clearance (MDRD) was 46 ml/min. Doppler echocardiography showed mainly kinetic disorders (89.3%) and left ventricular systolic dysfunction (71%). The three renal ultrasound examinations were normal. Six deaths (16.7%) were recorded.

**Conclusion:**

Cardiorenal syndrome is a reality and marks a turning point in the evolution of heart and kidney diseases. In Senegal, its prevalence in the Cardiology Department is low. Prospective multicentric studies should be conducted in order to better evaluate this syndrome in Senegal.

## Introduction

Le syndrome cardio-rénal (SCR) est un trouble physiopathologique du cœur et des reins dans lequel une dysfonction chronique ou aiguë de l’un peut induire une dysfonction chronique ou aiguë de l’autre. Le syndrome cardio-rénal (SCR) est classé par Ronco [1] en cinq sous-types (Tableau 1). Selon le registre américain ADHERE, au moins 65% des patients présentant un tableau d’insuffisance cardiaque aiguë ont une clairance de la créatinine < 60 ml/min [2]. L’étude CHARM a montré que 40% des patients en stade II et III de la New York Heart Association (NYHA) avaient une insuffisance rénale avérée [3]. En Afrique et au Sénégal en particulier l’incidence du syndrome cardio-rénal est mal connue. L’objectif de cette étude était d’apprécier la prévalence du SCR en milieu cardiologique sénégalais.

**Tableau 1:** Classification de RONCO du syndrome cardio-rénal

TYPES	CARACTERISTIQUES
**Type 1**	SCR aigu caractérisé par une insuffisance cardiaque aiguë décompensée qui conduit à l’insuffisance rénale aiguë.
**Type 2**	Le SCR chronique se caractérise par une insuffisance cardiaque chronique qui entraîne une maladie rénale chronique.
**Type 3**	Le syndrome réno-cardiaque aigu est dû à l'insuffisance rénale aiguë qui conduit à la dysfonction cardiaque aiguë, comme une arythmie ou une insuffisance cardiaque.
**Type 4**	Le syndrome réno-cardiaque chronique est caractérisé par une maladie rénale chronique primaire qui contribue à la dysfonction cardiaque.
**Type 5**	Aussi appelé SCR secondaire où il y a une atteinte simultanée rénale et cardiaque due à des troubles systémiques tels que la septicémie ou le lupus érythémateux systémique.

SCR: Syndrome cardio-rénal

## Méthodes

Nous avons mené une étude rétrospective incluant tous les patient insuffisants cardiaques ayant une altération de leur fonction rénale et hospitalisés entre Avril 2010 et avril 2011 au service de cardiologie du Centre Hospitalier Universitaire Aristide Le Dantec. Ces patients ont bénéficié d’un examen clinique complet et d’examens paracliniques. Les signes d’insuffisance cardiaque (IC) étaient la dyspnée (selon la NYHA), les râles crépitants, le bruit de galop gauche pour l’IC gauche et l’hépatalgie, le bruit de galop droit, l’hépatomégalie, les oedèmes des membres inférieurs, la turgescence des veines jugulaires pour l’IC droite. L’atteinte rénale était retenue pour une créatininémie supérieure à 12m chez la femme et 13 mg/L chez l’homme. Les données étaient analysées avec le logiciel statistique Epi-info 3.5.3.

## Résultats

**Figure 1:**
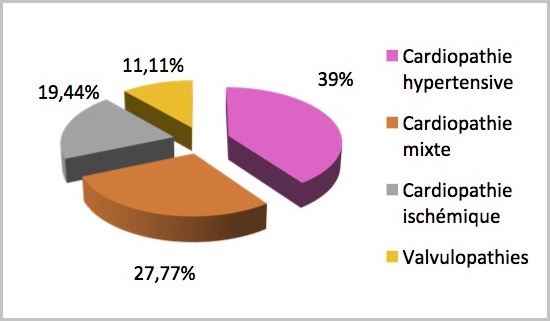
Répartition des patients en fonction des étiologies de l’insuffisance cardiaque

**Figure 2:**
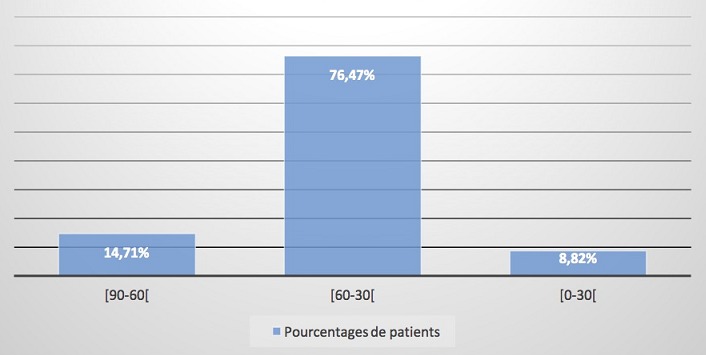
Répartition des patients atteints de syndrome cardio-rénal en fonction de la clairance de la créatininine (ml/min)

Nous avons inclus 36 patients. La prévalence hospitalière du SCR était de 3,7% avec une prédominance masculine (sex-ratio à 1,77). L’âge moyen était à 56,9 ans avec des extrêmes de 30 et 92 ans. Les tranches d’âge les plus représentatives étaient celles comprises entre (50-70 ans (54,6%) et (70-90) ans (25,9%). Les antécédents étaient dominés par l’hypertension artérielle avec 19 cas (52,78%) suivie du diabète (19,4%), de l’insuffisance cardiaque chez 8 patients (22,2%) et des valvulopathies (3 patients). En outre étaient retrouvés un trouble du rythme chez 2 patients (5,6%), une insuffisance rénale chronique, une hyperthyroïdie et une notion de phytothérapie dans un cas chacun. Les étiologies étaient la cardiopathie hypertensive (39%), l’insuffisance coronarienne (19,44%), la cardiopathie mixte (hypertensive et ischémique) (27,78%), la valvulopathie (11,11%), l’insuffisance rénale (2,78%) et la cardiothyréose (2,78%) (Figure 1). Les signes étaient représentés par un tableau d’insuffisance ventriculaire gauche, droite et globale respectivement dans 69,4% et 50% et 50% des patients. Les détails des signes cliniques sont représentés dans le Tableau 1. A la biologie, on notait une anémie chez 17 patients, qui était sévère chez 7 patients (19,4%) et modérée dans 10 cas (27,8%). La créatininémie moyenne était à 20,28 mg/l (13-63). Cette hypercréatininémie était majoritairement comprise entre (13-20) chez 24 malades (66,67%). La clairance de la créatinine était en moyenne à 46 ml/ min. Elle était comprise entre (90-60) chez 14,71%, (30-60)chez 76,47% et (0-30) pour 8,82% (Figure 2). Le syndrome cardio-rénal était majoritairement de type 2 (97,22%).

En outre on notait une hyponatrémie (68 > 22%).

En outre on notait une hyponatrémie (68,18%), une hypokaliémie (20,09%), une hyperleucocytose et une thrombopénie respectivement dans 9 (26,4%) et 5 cas (14,7%). L’électrocardiogramme inscrivait des anomalies dominées par l’hypertrophie auriculaire gauche (HAG) dans 16 cas (44,44 %), l’hypertrophie ventriculaire gauche (HVG) chez 12 patients (33,33 %), l’hémibloc antérieur chez 10 patients (27,78 %), l’hypertrophie ventriculaire droite (HVD) et la tachycardie sinusale régulière dans 8 cas chacun. La radiographie de face était réalisée chez 35 patients. Elle mettait en évidence une cardiomégalie chez 29 patients (80,55%). Le rapport cardio-thoracique moyen était de 0,55 [0,46-0,84]. Des signes d’hypertension veineuse pulmonaire étaient observés dans 48,5% (17 patients). L’échocardiographie Doppler retrouvait majoritairement des troubles de la cinétique (89,3%), une dysfonction du ventricule gauche systolique (71%) et diastolique (44,44%); une dilatation du ventricule droit, du ventricule gauche et de l’oreillette gauche respectivement dans 39,3%, 50% 71,4%. Une hypertension artérielle pulmonaire était notée chez 20 patients (55,55%). L’échographie rénale réalisée chez 3 malades était revenue normale. Le régime hyposodé était proposé chez 26 patients (72,2%). Les patient ont reçu des diurétiques dans 94,44% (34 patients), des inhibiteurs de l’enzyme de conversion chez 88,89% (32 patients), des inhibiteurs calciques chez 52,78% (19 patients) et des bêta-bloquants chez 9 patients (25%). L’évolution était favorable sous traitement avec régression des signes d’IC. Certaines complications ont été notées à type d’embolie pulmonaire (2 cas), de choc cardiogénique (2 patients), de flutter atrial (4 cas) et de fibrillation atriale (4 malades). La durée moyenne d’hospitalisation était de 28,12 jours [2-76]. Six décès (16,7 %) étaient notés.

## Discussion

La prévalence du SCR de l’ordre de 3,7% dans notre série est faible comparée à celle relatée dans la littérature, 40% dans l’étude CHARM (patients en stade II et III de la NYHA) et au moins 65% selon le registre américain ADHERE [2, 3]. Cette différence est en partie due au faible échantillon dans notre série, au caractère rétrospectif de l’étude mais surtout au retard du dosage biologique qui fait que la fonction rénale se normalise entre temps sous traitement. Notre population est relativement jeune avec un âge moyen de 56,9 ans comme dans la plupart des études africaines menées sur l’insuffisance cardiaque notamment Kingue avec 57,26 ± 16,04 ans [4]. Les âges de prédilection de cette pathologie étaient les tranches d’âge (50-70) ans (54,6%) et (70-90) ans (25,9%) dans nos pays alors qu’en Occident il s’agit d’une pathologie du sujet âgé avec un diagnostic autour de 70 ans [5, 6]. Il y avait une prédominance masculine (sex-ratio à 1,77) en phase avec Kingue (sex-ratio à 1,3) alors que Go avait une prédominance féminine à 55% [4, 7]. Les étiologies comme dans la plupart de la littérature (Ikama) restent dominées par la cardiopathie hypertensive (39%), la cardiopathie mixte (hypertensive et ischémique) (27,77%) et l’insuffisance coronarienne (19,44%) [8] alors que Thiam en 2000 à l’hôpital Principal de Dakar avait une prévalence plus élevée des valvulopathies dans l’insuffisance cardiaque, 44,7% contre 34,1 % pour la cardiopathie hypertensive en 2e position [9]. Ceci nous amène à constater une émergence des maladies chroniques en Afrique en particulier l’HTA et l’insuffisance coronaire. L’apparition du SCR sur une valvulopathie (11,11%) marque le caractère évolué de celle-ci posant ainsi le problème de leur prise en charge chirurgicale. Dans la majorité des cas (97,22%), le SCR était de type 2, l’étude étant menée dans un service de cardiologie où sont admis les insuffisants cardiaques. Dans notre série, le tableau d’insuffisance ventriculaire gauche et droite était retrouvé respectivement dans 69,4% et 50% des individus. A la biologie, l’atteinte rénale était constante avec une clairance de la créatinine moyenne à 46 ml/ min. Selon Volpe et Levine, le risque cardio-vasculaire est majoré dès que la fonction rénale est légèrement altérée, inférieure à 65 ml/min seulement [10, 11]. Silverberg dans son étude a montré que la sévérité de l’anémie est proportionnelle à la gravité de l’IC selon la classification de la NYHA [12]. La plupart de nos patients sont admis au stade IV (NYHA) du fait du retard à la consultation avec à l’échocardiographie une altération de la fonction systolique du VG chez 71% patients. L’atteinte rénale, en plus de la sévérité de l’IC, de l’anémie, explique la mortalité élevée de 16,67% ainsi que l’augmentation significative de la durée de séjour retrouvées dans notre étude [13–16].

## Conclusion

Le syndrome cardio-rénal est une réalité et marque un tournant dans l’évolution de toute cardiopathie et néphropathie. Sa prévalence en milieu cardiologique sénégalais est de 3,7% nettement inférieure à ce qui est rapporté dans la littérature. Des études prospectives et multicentriques sont nécessaires pour mieux déterminer la prévalence du SCR en Afrique et au Sénégal en particulier.

### Etat des connaissances actuelles sur le sujet

- Le syndrome cardio-rénal est une entité fréquente en milieu hospitalier cardiologique;
- Elle marque un tournant dans l’évolution de la cardiopathie;
- Le syndrome cardio-rénal de type 1 est plus fréquent en milieu cardiologique.

### Contribution de notre étude à la connaissance

- La prévalence retrouvée en milieu cardiologique sénégalais est faible;
- Il s’agit le plus souvent de syndrome cardio-rénal de type 2;
- Les étiologies sont dominées par les cardiopathies hypertensives et ischémiques.

## Conflits d’intérêts

Les auteurs ne déclarent aucun conflit d'intérêts.
